# Supplementary material for: The Role of Maladaptive Plasticity in Modulating Pain Pressure Threshold Post-Spinal Cord Injury
Source: Healthcare (Basel). 2025 Jan 26;13(3):247. doi: 10.3390/healthcare13030247 (PMC11816816; doi:10.3390/healthcare13030247)
Supplement: Supplementary file 1 [file healthcare-13-00247-s001.zip › Figure S4.pdf]

Figure S4: Sensitivity analysis PPT Bilateral

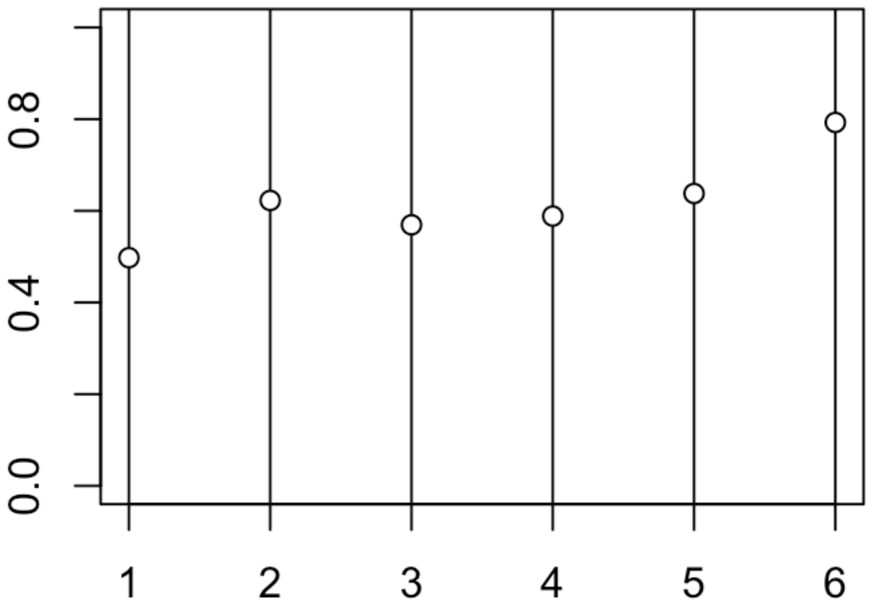

1: age; 2: sex; 3: etiology of lesion; 4: ASIA Impairment Scale; 5: EEG with the Low Beta activity in the central region Bilateral; 6: Handgrip Strength Test
