# Supplementary material for: The Role of Maladaptive Plasticity in Modulating Pain Pressure Threshold Post-Spinal Cord Injury
Source: Healthcare (Basel). 2025 Jan 26;13(3):247. doi: 10.3390/healthcare13030247 (PMC11816816; doi:10.3390/healthcare13030247)
Supplement: Supplementary file 1 [file healthcare-13-00247-s001.zip › Table S1.pdf]

Table S1: Supplementary Demographic Characteristics	*Participants n=102*
Marital Status	
Single	43 (47.3%)
Married	36 (39.6%)
Divorced	10 (11%)
Widowed	2 (2.2%)
Employment Status	
Employed	22 (24.2%)
Unemployed	21 (23.1%)
Away From Work	35 (38.5%)
Retired	13 (14.3%)
Smoking	
No	81 (89%)
Yes	10 (11%)
Alcohol Consumption	
No	60 (65.9%)
Yes	31 (34.1%)
Type of Incapacity	
Tetraplegia	45 (44.12%)
Paraplegia	57 (55.88%)
Dominance of side	
Right	85 (93.4%)
Left	6 (6.6%)
Comorbidities	
Chronic Pain	45 (44.18%)
Spasticity	35 (34.31%)
Neurogenic Bladder	28 (27.45%)
Arterial Hypertension	19 (18.63%)
Depression/ Insomnia	12 (11.77%)
Diabetes	10 (9.8%)
Dyslipidemia	10 (9.8%)
Osteoporosis	8 (7.84%)
Venous Thrombosis	5 (4.9%)
Medications	
Anticonvulsants	50 (49.02%)
Muscle Relaxant	41 (40.2%)
Antidepressants	35 (34.31%)
Antihypertensive	30 (29.41%)
Oxybutynin	22 (21.57%)

Ant glycemic	13 (12.75%)
Statins	13 (12.75%)
Opioid	3 (2.94%)
AINES	3 (2.94%)
MRC (Range 0-5)	
Upper Limb (Left)	4.41 (0.17)
Upper Limb (Right)	4.35 (0.22)
Lower Limb (Left)	1.88 (0.15)
Lower Limb (Right)	1.82 (0.17)
Pinch Pressure Test (kPa)	
Left Hand	7.7 (3.61)
Right Hand	7.32 (3.3)
Purdue Pegboard Test (sec)	
Left Hand	45.43 (53.16)
Right Hand	46.75 (67.94)

MRC: Medical Research Council Scale; kPa: Kilopascal; Sec: seconds

Continuous variables: mean (SD); Categorical variables: n(percentage)
