# Supplementary material for: The Role of Maladaptive Plasticity in Modulating Pain Pressure Threshold Post-Spinal Cord Injury
Source: Healthcare (Basel). 2025 Jan 26;13(3):247. doi: 10.3390/healthcare13030247 (PMC11816816; doi:10.3390/healthcare13030247)
Supplement: Supplementary file 1 [file healthcare-13-00247-s001.zip › Table S3.pdf]

Table S3: PPT Right thenar region	
Minimum	3.1 kPa
First Quartile	6.15 kPa
Median	8.32 kPa
Mean	8.59 kPa
Third Quartile	10.71 Kpa
Maximum	15.93 kPa
