# Supplementary material for: The Role of Maladaptive Plasticity in Modulating Pain Pressure Threshold Post-Spinal Cord Injury
Source: Healthcare (Basel). 2025 Jan 26;13(3):247. doi: 10.3390/healthcare13030247 (PMC11816816; doi:10.3390/healthcare13030247)
Supplement: Supplementary file 1 [file healthcare-13-00247-s001.zip › Table S5.pdf]

Table S5: Univariate PPT left side				
Variable	*β-coefficient*	*p value*	*std. error*	*adjusted r squared*
Primary lesion level non-cervical	2.3953	4.09115E-05	0.5548	0.1639
Tetraplegia	-2.2619	0.0001	0.5621	0.1444
Handgrip Strength Test Left side	0.0673	0.0001	0.0166	0.1808
Divorced	-3.1602	0.0018	0.9827	0.0789
ASIA Impairment Scale Incomplete	-2.0346	0.0025	0.6491	0.1040
Medical Research Council Scale Upper limb left	0.9379	0.0070	0.3396	0.0693
EEG region Frontal right Low Beta	-17.4491	0.0097	6.5858	0.0692
EEG region Frontal left Low Beta	-17.0282	0.0109	6.5303	0.0668
EEG region Frontal bilateral Low Beta	-16.8096	0.0122	6.5565	0.0644
EEG region Parietal right Low Beta	-14.2813	0.0192	5.9737	0.0557
Sensitive function test upper limbs altered	3.9490	0.0193	1.6565	0.0495
EEG region Parietal bilateral Low Beta	-14.8470	0.0228	6.3931	0.0514
EEG region Parietal left Low Beta	-13.3981	0.0264	5.9243	0.0483
EEG region Parietal right Beta	-9.3476	0.0290	4.2035	0.0464
Purdue Pegboard Test left	-0.0131	0.0292	0.0059	0.0528
EEG region Frontal left Beta	-9.2728	0.0301	4.1988	0.0457
EEG region Frontal right Beta	-9.3922	0.0315	4.2901	0.0447
EEG region Frontal bilateral Beta	-9.3730	0.0318	4.2885	0.0445
Years of education	-0.1446	0.0352	0.0676	0.0391
Sensitive function test upper limb right altered	4.2511	0.0392	2.0314	0.0362
EEG region Central right Beta	-7.6021	0.0396	3.6338	0.0400
EEG region Central bilateral Low Beta	-12.0589	0.0409	5.8033	0.0393
EEG region Central right Low Beta	-11.3148	0.0422	5.4784	0.0392
EEG region Central left Low Beta	-11.4301	0.0437	5.5760	0.0380
Pinch Strength Test left	0.1996	0.0453	0.0978	0.0451
EEG region Parietal bilateral Beta	-8.5046	0.0573	4.4089	0.0325
EEG region Parietal left Beta	-7.6419	0.0693	4.1512	0.0286
EEG region Central bilateral Beta	-6.9148	0.0749	3.8316	0.0271
Montreal Cognitive Assessment	-0.1424	0.0841	0.0812	0.0301
EEG region Central left Beta	-5.7311	0.1186	3.6331	0.0180
EEG region Central right High Beta	-13.3685	0.1253	8.6285	0.0172
Presence of neuropathic pain	-0.9448	0.1285	0.6158	0.0148
BMI (kg/m2) 25-29.99	1.6505	0.1304	1.0807	-0.0066
Pinch Strength Test right	0.1402	0.1368	0.0931	0.0189
EEG region Parietal High Beta	-14.9152	0.1888	11.2523	0.0094
BMI (kg/m2) 18.5-24.99	1.3350	0.1993	1.0322	-0.0066
