# Supplementary material for: The Role of Maladaptive Plasticity in Modulating Pain Pressure Threshold Post-Spinal Cord Injury
Source: Healthcare (Basel). 2025 Jan 26;13(3):247. doi: 10.3390/healthcare13030247 (PMC11816816; doi:10.3390/healthcare13030247)
Supplement: Supplementary file 1 [file healthcare-13-00247-s001.zip › Table S6.pdf]

Table S6: Univariate PPT right side				
Variable	*β-coefficient*	*p value*	*std. error*	*adjusted r squared*
ASIA Impairment Scale Incomplete	-2.2790	0.0007	0.6434	0.1304
Primary lesion level non-cervical	1.9855	0.0011	0.5865	0.1031
Handgrip Strength Test Right side	0.0533	0.0032	0.0175	0.1061
Tetraplegia	-1.7345	0.0045	0.5959	0.0759
Handgrip Strength Test Left side	0.0471	0.0119	0.0182	0.0749
Medical Research Council Scale Upper limb left	0.7432	0.0182	0.3090	0.0505
Purdue Pegboard Test left	-0.0140	0.0256	0.0061	0.0559
Years of education	-0.1550	0.0274	0.0691	0.0433
Divorced	-2.0198	0.0558	1.0420	0.0118
Functional Independence Measure	0.0245	0.0642	0.0131	0.0268
BMI (kg/m2) 25-29.99	2.0017	0.0726	1.1013	0.0070
EEG region Frontal bilateral Theta	7.1752	0.0819	4.0736	0.0250
Sensitive function test upper limb right altered	3.6556	0.0851	2.0999	0.0218
EEG region Frontal left Theta	7.3149	0.0879	4.2342	0.0236
Purdue Pegboard Test right	-0.0082	0.0928	0.0048	0.0258
EEG region Parietal bilateral Theta	7.0987	0.0932	4.1794	0.0225
EEG region Frontal right Low Beta	-11.5402	0.0941	6.8123	0.0223
EEG region Frontal left Delta	7.8351	0.0990	4.6947	0.0213
EEG region Parietal left Delta	8.1496	0.1119	5.0700	0.0189
BMI (kg/m2)	0.0991	0.1136	0.0620	0.0170
EEG region Frontal bilateral Delta	8.1081	0.1148	5.0858	0.0185
EEG region Frontal left Low Beta	-10.7456	0.1158	6.7600	0.0183
EEG region Parietal right Theta	6.5664	0.1179	4.1548	0.0179
EEG region Parietal bilateral Low Beta	-10.2439	0.1232	6.5754	0.0171
EEG region Frontal bilateral Low Beta	-10.5471	0.1237	6.7805	0.0170
EEG region Parietal left Theta	6.3563	0.1244	4.0939	0.0169
EEG region Central bilateral Low Beta	-9.1116	0.1285	5.9328	0.0163
EEG region Central left Low Beta	-8.7414	0.1289	5.6977	0.0162
EEG region Parietal left Low Beta	-9.2317	0.1328	6.0801	0.0157
EEG region Frontal right Theta	6.3559	0.1378	4.2404	0.0150
EEG region Parietal bilateral Delta	7.6910	0.1436	5.2074	0.0142
EEG region Central right Delta	7.5754	0.1454	5.1532	0.0140
EEG region Central right Low Beta	-8.1275	0.1501	5.5937	0.0135
EEG region Parietal right Low Beta	-8.9417	0.1508	6.1645	0.0134
EEG region Parietal right Beta	-6.1965	0.1549	4.3155	0.0128
EEG region Central right Beta	-5.3062	0.1565	3.7103	0.0126
Female	-1.3570	0.1587	0.9547	0.0112
EEG region Frontal right Beta	-6.1099	0.1687	4.3990	0.0112

EEG region Central bilateral Theta	5.8611	0.1784	4.3179	0.0102
EEG region Parietal right Delta	6.7800	0.1853	5.0753	0.0095
EEG region Parietal bilateral Beta	-5.9729	0.1890	4.5086	0.0091
EEG region Frontal left Beta	-5.6695	0.1924	4.3133	0.0088
EEG region Frontal bilateral Beta	-5.7404	0.1959	4.4016	0.0085
