# Supplementary material for: The Role of Maladaptive Plasticity in Modulating Pain Pressure Threshold Post-Spinal Cord Injury
Source: Healthcare (Basel). 2025 Jan 26;13(3):247. doi: 10.3390/healthcare13030247 (PMC11816816; doi:10.3390/healthcare13030247)
Supplement: Supplementary file 1 [file healthcare-13-00247-s001.zip › Table S7.pdf]

Table S7: Univariate PPT Bilateral

Variable	*β-coefficient*	*p value*	*std. error*	*adjusted r squared*
Primary lesion level non-cervical	2.2062	0.0001	0.5382	0.1480
Handgrip Strength Test Bilateral	0.0639	0.0001	0.0159	0.1741
Handgrip Strength Test Right side	0.0601	0.0003	0.0157	0.1631
Tetraplegia	-2.0149	0.0004	0.5471	0.1213
ASIA Impairment Scale Incomplete	-2.1775	0.0006	0.6108	0.1320
Handgrip Strength Test Left side	0.0572	0.0007	0.0162	0.1414
Medical Research Council Scale Upper limb left	0.8009	0.0066	0.2878	0.0697
Divorced	-2.5514	0.0097	0.9647	0.0445
Purdue Pegboard Test left	-0.0136	0.0187	0.0056	0.0633
Years of education	-0.1499	0.0234	0.0650	0.0463
EEG region Frontal right Low Beta	-14.3249	0.0267	6.3472	0.0475
EEG region Frontal left Low Beta	-13.7158	0.0323	6.2984	0.0436
EEG region Frontal bilateral Low Beta	-13.4848	0.0359	6.3205	0.0415
EEG region Parietal bilateral Low Beta	-12.5826	0.0438	6.1424	0.0375
Sensitive function test upper limb right altered	3.9676	0.0466	1.9662	0.0327
EEG region Parietal left Low Beta	-11.4395	0.0473	5.6802	0.0359
EEG region Parietal right Low Beta	-11.5100	0.0488	5.7536	0.0357
EEG region Parietal right Beta	-7.6530	0.0615	4.0367	0.0307
EEG region Central bilateral Low Beta	-10.5283	0.0618	5.5598	0.0306
EEG region Central left Low Beta	-10.0998	0.0621	5.3396	0.0305
EEG region Frontal right Beta	-7.5840	0.0691	4.1165	0.0284
EEG region Central right Low Beta	-9.5152	0.0733	5.2432	0.0275
EEG region Frontal left Beta	-7.3116	0.0736	4.0340	0.0271
EEG region Central right Beta	-6.2825	0.0745	3.4771	0.0269
EEG region Frontal bilateral Beta	-7.3781	0.0769	4.1177	0.0262
BMI (kg/m2) 25-29.99	1.8261	0.0826	1.0397	0.0018
Pinch Strength Test bilateral	0.1641	0.0851	0.0939	0.0293
Functional Independence Measure	0.0214	0.0870	0.0124	0.0214
Sensitive function test upper limbs altered	3.0642	0.0895	1.7852	0.0209
EEG region Parietal bilateral Beta	-7.2363	0.0907	4.2261	0.0230
EEG region Frontal bilateral Theta	6.3708	0.1022	3.8539	0.0207
EEG region Parietal left Beta	-6.4838	0.1066	3.9732	0.0199
EEG region Frontal left Theta	6.3388	0.1177	4.0087	0.0180
EEG region Central bilateral Beta	-5.7915	0.1185	3.6707	0.0178
Pinch Strength Test left	0.1459	0.1265	0.0943	0.0204
EEG region Parietal bilateral Theta	5.9265	0.1384	3.9606	0.0149
EEG region Parietal left Delta	6.9621	0.1508	4.7999	0.0133
EEG region Parietal left Theta	5.4218	0.1656	3.8751	0.0115

Pinch Strength Test right	0.1235	0.1658	0.0881	0.0144
EEG region Frontal right Theta	5.4792	0.1758	4.0121	0.0104
EEG region Frontal bilateral Delta	6.5023	0.1813	4.8219	0.0099
EEG region Central left Beta	-4.6901	0.1815	3.4799	0.0099
EEG region Parietal right Theta	5.3030	0.1819	3.9383	0.0098
BMI (kg/m2)	0.0777	0.1887	0.0586	0.0083
EEG region Parietal bilateral Delta	6.4921	0.1915	4.9290	0.0089
