# Supplementary figures and images for: The Role of Maladaptive Plasticity in Modulating Pain Pressure Threshold Post-Spinal Cord Injury
Source: Healthcare (Basel). 2025 Jan 26;13(3):247. doi: 10.3390/healthcare13030247 (PMC11816816; doi:10.3390/healthcare13030247)

Figure S1: Distribution of PPT Left thenar region

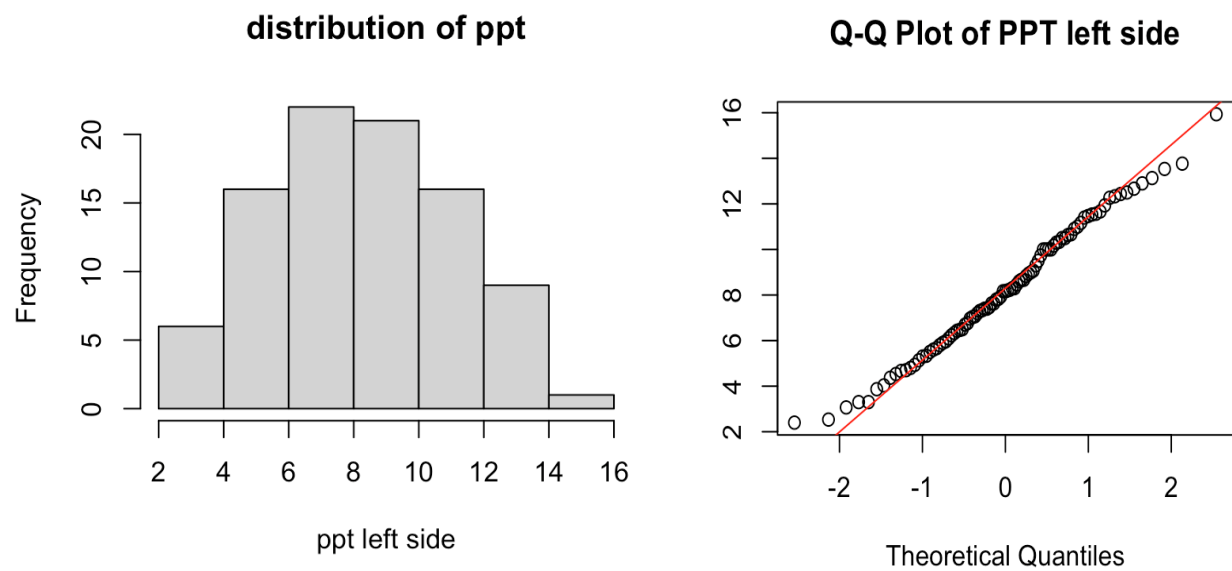

Supplement: Supplementary file 1 [file healthcare-13-00247-s001.zip › Figure S1.pdf]

Figure S2: Distribution of PPT Right thenar region

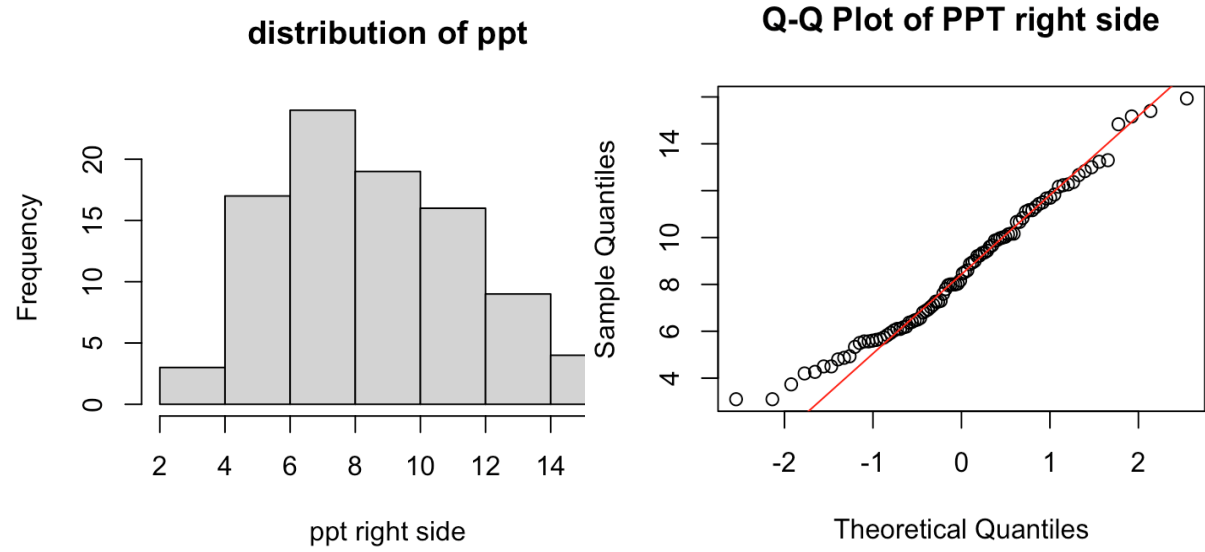

Supplement: Supplementary file 1 [file healthcare-13-00247-s001.zip › Figure S2.pdf]

Figure S3: Distribution of PPT Bilateral of thenar regions

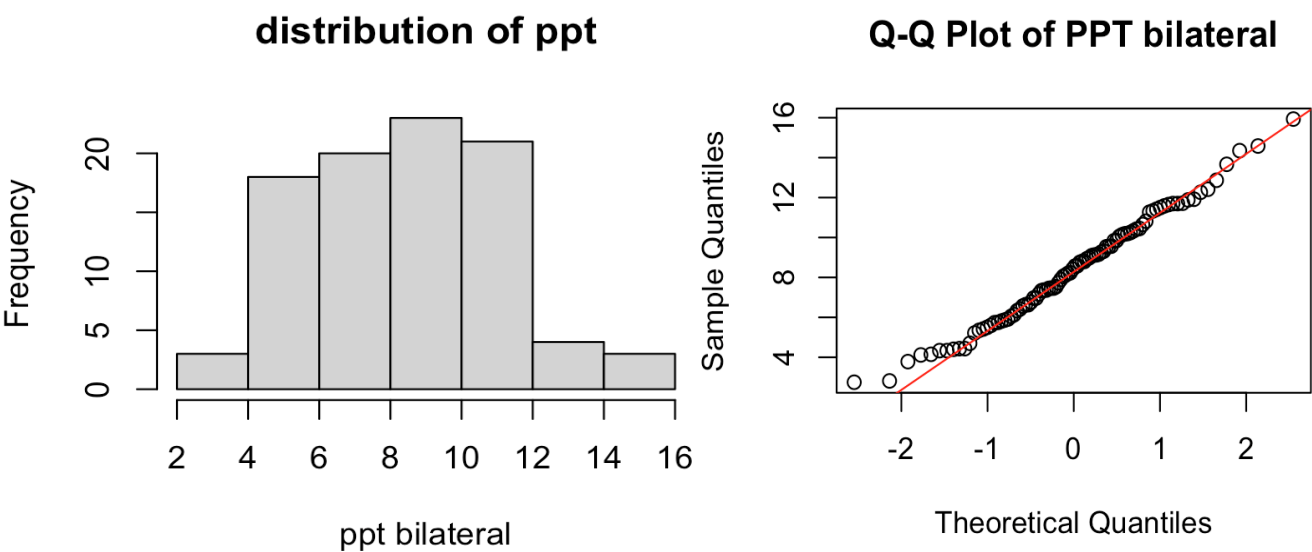

Supplement: Supplementary file 1 [file healthcare-13-00247-s001.zip › Figure S3.pdf]
